# Does Smartphone Use Really Impact Cervical Rotation and Cervical Proprioception in Asymptomatic Individuals?

**DOI:** 10.7759/cureus.37170

**Published:** 2023-04-05

**Authors:** Aishwarya A Pashine, Simran Jethani, Shilpa Chourasia

**Affiliations:** 1 Department of Physiotherapy, VSPM's College of Physiotherapy, Nagpur, IND; 2 Department of Musculoskeletal Physiotherapy, VSPM's College of Physiotherapy, Nagpur, IND

**Keywords:** neck disability index, cervical proprioception, cervical rotation, asymptomatic population, smartphone addiction

## Abstract

Background and objective

Smartphone use has increased exponentially over the last two years worldwide. The outbreak of the coronavirus disease 2019 (COVID-19) pandemic led to significantly higher dependence on the smartphone for information exchange and communication among the general public. Currently, India has hundreds of millions of smartphone users, and their numbers are on the rise. This has raised concerns regarding the adverse effects of smartphone use on mental and musculoskeletal health. In light of this, this study aimed to determine and evaluate the musculoskeletal consequences of smartphone use.

Method

A total of 102 participants (50 adolescents and 52 adults) who were smartphone users and are asymptomatic for cervical spine-related disorders were included based on convenience sampling. The components assessed were cervical rotation using tape measurement and cervical proprioception using the head repositioning accuracy test. Frequency distribution tables and text were used to report the results.

Results

The results of this research indicated reduced cervical rotation range and cervical proprioception deficits in both adolescent and adult smartphone users. Furthermore, no correlation was found between cervical rotation (right and left) and cervical proprioception (right and left rotation).

Conclusion

Though the results showed that both the individual components - cervical rotation and cervical proprioception - were significantly affected, there was no correlation between the components, which indicates that these asymptomatic individuals who are marginally excessive smartphone users are at increased risk for reduced cervical mobility and deficits in cervical proprioception.

## Introduction

Smartphones have become the principal source of communication and information exchange in our daily lives. Smartphones have gained immense popularity over the last few years due to their ability to provide social networking services, internet access, and access to smartphone applications [1]. About 45.12% of the global population possess a smartphone. With regard to the penetration of smartphones among the Indian population, while there were only 291.6 million smartphone users in 2017, it was expected to reach 490.9 million by 2022 [2]. The excessive of smartphones has raised concerns about its potential implications on safety and health, including mental and musculoskeletal health [1]. Several studies suggest that long-term smartphone use results in sustained poor posture, which imposes physiological loads on the neck that deviate from the normal state, thereby compromising the functioning of the cervical spine [3].

Head and neck movements are crucial elements of our daily interaction with the world [4]. Reduced active movement in the cervical region has been linked to alterations in the passive components of the cervical spine, which can result in various consequences that involve disruption of operational activities, impaired defensive and restorative responses, and diminished neck mobility [5]. Since cervical axial rotation is one of the most frequently used movements in daily activities, it deserves particular attention while evaluating cervical function [4].

A highly complex proprioceptive system controls the cervical spine, the vertebral column's most mobile segment [6]. The umbrella term "proprioception" refers to non-visual information pertaining to the awareness of the body in space. Normal motor control and motor learning depend heavily on proprioception [7]. The sensorimotor extent of the cervical spine is immensely affected due to the deterioration of neck proprioception, which has a direct impact on postural control and balance [8]. Any anatomical and functional alterations in the cervical deep and superficial muscles, which impact afferent inputs and change proprioception, may cause variations in the discharge of muscle spindles [9].

In terms of addictive behaviors, the vast bulk of studies focuses on drug use disorders, whereas the addictive internet-related behaviors caused by the ongoing coronavirus disease 2019 (COVID-19) pandemic have received relatively little attention. About 8.4%-24.9% of adolescents and 52.9% of young adults are reported to be smartphone overusers [10]. The cervical range of motion is the most accurate and standardized functional measurement to assess cervical spine function [4]. Whereas, cervical proprioception is important for motor learning, motor control, and maintenance of balance and posture. As this research was conducted among asymptomatic individuals in the age group of 10-30 years, a demographic that is particularly vulnerable to smartphone use, we focused on assessing the commonly affected structure - the cervical spine - in terms of rotation range and proprioception as well as evaluating the correlation between them [7].

Diverse studies have examined the cervical spine's alignment, the cervical region's active range of motion, and the cervical proprioception in symptomatic individuals, i.e., those with a Neck Disability Index (NDI) score of more than 5 [11], or among excessive smartphone users with a Smartphone Addiction Scale - Short Version (SAS-SV) score of more than 30, with the receiver operating characteristic (ROC) curve analysis results of an area under the curve (AUC) value of 0.947 (0.887-1.000), a cut-off value of 33, sensitivity of 0.875, and specificity of 0.886 in females, and AUC value of 0.963 (0.888-1.000), a cut-off value of 31, sensitivity of 0.867, and specificity of 0.893 in males [12,13]. However, there is a dearth of studies in the literature on changes in cervical rotation and cervical proprioception in the asymptomatic population, which prompted us to conduct this study.

## Materials and methods

Study design and setting

This was an assessment-based study performed after receiving requisite approval from the Head of Institution and Institutional Ethics Committee (IEC) with IEC number (VSPMSCOP/UG-2021SUMMER01). The study participants signed informed consent that was obtained after a brief description of the goal, purpose, advantages, and shortcomings of the investigation. Participants answered questions on age, gender, hand dominance, NDI, and the SAS-SV. Healthy individuals from within and outside the institute were enlisted based on convenience sampling from December 2021 to May 2022. Overall, 102 participants aged 10-30 years were recruited for the study, out of which 50 were adolescents and 52 were adults. The participants were smartphone users with NDI scores of less than 5. Participants with NDI scores of more than 5, those with a history of headaches and migraines, any dysfunction associated with the cervical region, history of cervical radiculopathy or myelopathy, and those with vestibular disorders, any sensorimotor disease, or tumors or inflammation of the cervical spine were excluded from the study.

Methodology

Measuring equipment and materials such as measuring tape, graph paper, helmet fixed with laser pointer, calculator, stool/chair, and marker were used to record the findings. The cervical rotation range was assessed with tape measurements [14,15] and cervical proprioception was assessed using a head repositioning accuracy test.

The assessment of cervical rotation was followed by an assessment of cervical proprioception with a head repositioning accuracy test in which the individual was made to sit upright on a stool at a distance of 90 cm. A laser beam is then centered on the (0.0) point on the wall graph chart and then the participant was instructed to close the eyes and flex the head maximally and bring the head back to the perceived neutral (0.0) point. The study investigator marked the location of the laser beam on the graph paper and moved the head to the (0.0) position. The process was carried out three times, and the average distance was computed each time. For the remaining five neck movements, the process was repeated [16,17], and no participants reported experiencing any pain or neurological symptom related to cervical spine disorders. The X and Y values for each study participant were recorded in centimeters and entered into a Microsoft Excel spreadsheet for further analysis. Every point marked on graph paper had a distinct X and Y coordinate (Cartesian coordinate system). All precautions were taken to guarantee the privacy of data both during and after data collection.

Statistical analysis

Epi Info software version 7 was used to code and analyze the data once it was entered into MS Excel. Quantitative variables with mean and standard deviation (SD) and qualitative variables with frequency and percent were summarized by descriptive statistics. Interferential statistics included a test of significance and p-value. An unpaired t-test was used to find significance for individual components of cervical rotation (right and left) and cervical proprioception for all six cervical movements (flexion, extension, right rotation, left rotation, right lateral flexion, and left lateral flexion). Right and left cervical rotations were linked to right and left cervical proprioception respectively by using Fisher's exact test. Cervical rotation on the right and left sides and cervical proprioception on the right and left sides respectively were correlated using Pearson's correlation. A p-value <0.05 was considered statistically significant.

## Results

The mean SAS-SV score was found to be 30.71 ±8.32, which indicates that the participants were marginally excessive smartphone users. The normal range of the SAS-SV score is 0-30 (non-excessive smartphone users), and a score of 30-60 indicates excessive smartphone use [12,13].

The normal values for cervical rotation are between 11 to 13.2 cm [14,15], which is the difference between the starting position value (head in neutral position) and end position value (head in right or left rotation). The mean values for cervical rotation of the right and left side were 9.46 ±1.72 and 9.42 ±1.72 respectively, which indicates that left-side cervical rotation was more affected than right-side cervical rotation when compared with the normal values (Table 1).

**Table 1:** Mean cervical rotation

	Mean (right)	Mean (left)
Cervical rotation	9.46 cm	9.42 cm
Std. deviation	1.72	1.72
P-value	<0.0012	<0.008

The normal head repositioning error distance for cervical proprioception is 0-5 cm [18]. The mean values for errors in the distance indicate that right lateral flexion was more affected in proprioception followed by flexion, left lateral flexion, left rotation, extension, and right rotation (Table 2) when compared with the normal values of head repositioning error distance.

**Table 2:** Mean cervical proprioception

Cervical proprioception	N	Mean	Std. deviation	P-value
Flexion	102	7.043	3.489	<0.0001
Extension	102	7.31	3.39	<0.0001
Right rotation	102	7.911	3.80	<0.0001
Left rotation	102	7.2	3.57	<0.0001
Right lateral flexion	102	6.88	3.52	<0.0001
Left lateral flexion	102	7.123	3.32	<0.0001

The crosstabulation for right cervical rotation and right cervical proprioception demonstrated that out of 102 participants, 77 (75.49%) showed reduced ranges of right-side cervical rotation, and 88 (86.27%) participants showed right-side cervical proprioception deficits (Table 3).

**Table 3:** Crosstabulation for right-side cervical rotation and right-side cervical proprioception

			Right cervical proprioception (cm)		Total
			Affected	Normal	
Right cervical rotation (cm)	Affected	Count	65	12	77
		% within right cervical proprioception (cm)	63.72%	11.76%	75.49%
	Normal	Count	23	2	25
		% within right cervical proprioception (cm)	22.54%	1.9%	24.45%
Total		Count	88	14	102
		% within right cervical proprioception (cm)	86.27%	13.72%	100.0%

The crosstabulation for left cervical rotation and left cervical proprioception demonstrated that out of 102 participants, 77 (75.5%) showed reduced ranges of left-side cervical rotation, and 85 (83.33%) participants showed left-side cervical proprioception deficits (Table 4).

**Table 4:** Crosstabulation for left-side cervical rotation and left-side cervical proprioception

			Left cervical proprioception (cm)		Total
			Affected	Normal	
Left cervical rotation (cm)	Affected	Count	63	14	77
		% within left cervical proprioception (cm)	61.76%	13.72%	75.5%
	Normal	Count	22	3	25
		% within left cervical proprioception (cm)	21.5%	2.94%	24.5%
Total		Count	85	17	102
		% within left cervical proprioception (cm)	83.33%	16.66%	100.0%

When both the components of right cervical rotation (difference of ranges in cm) and right cervical proprioception (head repositioning error distance in cm) were correlated using Pearson's correlation, the p-value came out to be 0.2679, which was statistically not significant and suggests that there was no correlation between right cervical rotation and right cervical proprioception (Table 5).

**Table 5:** Correlation between right-side cervical rotation and right-side cervical proprioception

		Right cervical proprioception (cm)
Right cervical rotation (cm)	Pearson's correlation	-0.1107
	P-value	0.2679
	N	102

When both the components of left cervical rotation (difference of ranges in cm) and left cervical proprioception (head repositioning error distance in cm) were correlated using Pearson's correlation, the p-value came out to be 0.4240, which was statistically not significant and suggests that there was no correlation between left cervical rotation and left cervical proprioception (Table 6).

**Table 6:** Correlation between left-side cervical rotation and left-side cervical proprioception

		Left cervical proprioception (cm)
Left cervical rotation (cm)	Pearson's correlation	-0.08
	P-value	0.4240
	N	102

## Discussion

The current study focused on correlating the ranges of cervical rotation on the right and left sides with cervical proprioception deficits of right and left cervical rotations in smartphone users aged 10-30 years. The mean SAS-SV score was 30.71, which indicates that the participants were marginally excessive smartphone users (SAS-SV: 0-30 - non-excessive; 30-60 - excessive smartphone users). Smartphones can lead to poor posture, including slumped or forward head posture (FHP), as their smaller screens force users to look down or extend their arms out in front of them to see the screen.

The neck rotation test is used to assess cervical spine dysfunction. Regulating the stimulation of the neck muscles is crucial for appropriate cervical rotation involving a complete range of motion for both the right and left sides without restrictions. However, accurate neck movement with an improper posture population is difficult because it is associated with problems with alteration in length and stimulation of the neck muscles. Previous research has revealed that the FHP group had significantly less axial neck rotation compared to the general population [19]. This is consistent with the findings of the present study for cervical rotation, which showed reduced ranges for both right and left cervical rotation regardless of hand dominance, which indicates an increased risk of cervical spine-related disorders. These results are in line with the findings of Samir et al. who deduced that due to extensive smartphone use, the cervical range of motion was decreased, which is related to the fact that prolonged time spent hunched over a smartphone can adversely affect posture without any obvious symptoms [12]. The weights put cervical-sensitive tissues at risk, affecting the function and range of motion of the cervical spine, thereby causing a musculoskeletal imbalance in the upper body [3].

The second component that we assessed was cervical proprioception, and based on our findings, the mean head reposition error distance of cervical proprioception was more affected by right lateral flexion, followed by flexion, left lateral flexion, left rotation, extension, and then right rotation. The normal range for cervical proprioception is 0-5 cm, and a value of more than 5 cm is considered a cervical proprioception deficit. In this study, cervical proprioception deficit for all six cervical movements revealed a p-value <0.0001, which is considered extremely significant. When compared to Lee et al.’s research, which showed significant joint position error (JPE) in right rotation followed by flexion, extension, and left rotation, the overall mean JPE in this study was more substantial. The possible reason for this is that our subjects were marginally excessive smartphone users, meaning that they adopt a more static neck posture, resulting in a more head repositioning error distance [16]. A study by Teng et.al., which compared middle-aged adults with neck discomfort, middle-aged participants without neck pain, and a young asymptomatic group in terms of head repositioning accuracy as a measure of cervical spine proprioception, concluded that repositioning mistakes were greater in both older groups, indicating that aging affected cervical proprioception. More studies involving the younger population are required since they are more prone to experiencing "text neck" due to their increased use of mobile devices, which is the main strength of this study. People who use unsupported mobile electronic gadgets for extended periods are said to have "text neck," which refers to neck pain [20]. According to Kim et al., the neck region was the area of the body with the most pain. They also reported that long and continuous smartphone use can cause proprioceptive deficits in cervical vertebrae [19].

Holding a forward head posture for an extended period changes cervical motor control and makes neck muscles such as the sternocleidomastoid unstable. Alterations in the sternocleidomastoid's activation may result in aberrant neck rotational biomechanics. Neck movements have been linked to improper alignment, neck muscle instability, and altered cervical spine motor control [21]. A previous study has shown that deteriorated muscle spindle afferent transmission brought on by tiredness, illness, or aging has an impact on proprioception [22]. When attempting to move to the desired head position, different motor methods may be used by people, which may account for the rise in the head repositioning error distance [23]. Lee et al. have reported that prolonged smartphone use affects cervical proprioception in young and asymptomatic individuals. They also concluded that regular smartphone use may lead to soft tissue distortion in the cervical spine, including the sternocleidomastoid, cervical erector spinae, and upper trapezius, which may exacerbate repositioning errors [6,24].

However, when both the components of right cervical rotation and right cervical proprioception were correlated in this study, the results were statistically not significant and suggested no correlation between cervical rotation and cervical proprioception for both sides. That the association of coefficients was not strong enough could be due to the fact that participants were an asymptomatic group. A population with symptoms would show a stronger association. This study involved a younger population of 10 to 30 years of age who were marginally excessive smartphone users and showed reduced cervical right and left rotation ranges with increased head reposition error distance (cervical proprioception deficits), which suggests that these individuals are at a higher risk for neck disabilities and smartphone addiction-related consequences. Additionally, our sample size was relatively small and we assessed only two components of the cervical movements, which represent two major limitations of this study. Future research involving more cervical movement components and a larger sample size is certainly warranted.

Programs to raise awareness about the effectiveness of cervical proprioception in the prevention and treatment of cervical illnesses need to be established, as there is currently a lack of awareness in this regard. A critical component of rehabilitation related to the process of repairing major joints is proprioceptive training, and, subject to further research, it may be used in cervical joint rehabilitation [25]. Our findings suggest that when using a smartphone, young people need to be aware of their posture and make efforts to correct incorrect neck alignment. The young generation can be made aware of their poor posture by providing information on mobile applications like Text Neck® Indicator [20]. This can help reduce the likelihood of developing chronic severe neck pain and related cervical problems in the future.

## Conclusions

This study concludes that marginally excessive smartphone users in the age group of 10-30 years showed a significant restriction in cervical rotation ranges for both right and left sides as well as an increase in head repositioning error distance for all six movements - flexion, extension, right rotation, left rotation, right lateral flexion, and left lateral flexion. Furthermore, the present research found no correlation between cervical rotation and cervical proprioception, which indicates that these asymptomatic individuals who are marginally excessive smartphone users are at increased risk of reduced cervical mobility and deficits in cervical proprioception. Hence, maintenance of appropriate posture while using a smartphone should be prioritized to avoid postural instability leading to musculoskeletal disorders in the future.
